# Is paternal oxytocin an oxymoron? Oxytocin, vasopressin, testosterone, oestradiol and cortisol in emerging fatherhood

**DOI:** 10.1098/rstb.2021.0060

**Published:** 2022-08-29

**Authors:** Marian J. Bakermans-Kranenburg, Martine W. F. T. Verhees, Anna M. Lotz, Kim Alyousefi-van Dijk, Marinus H. van IJzendoorn

**Affiliations:** ^1^ Department of Clinical Child and Family Studies, and Amsterdam Public Health, Vrije Universiteit Amsterdam, 1085 BT, Amsterdam, The Netherlands; ^2^ Leiden Consortium on Individual Development, 2300 RB, Leiden, The Netherlands; ^3^ Quantitative Psychology and Individual Differences, KU Leuven, Leuven, Belgium; ^4^ Research Department of Clinical, Educational and Health Psychology, Faculty of Brain Sciences, UCL, University of London, London W1T 7NF, UK

**Keywords:** fathers, hormones, parenting, perinatal, sensitivity, pregnancy

## Abstract

How do hormonal levels in men change from pregnancy to after the birth of their firstborn child, and what is the role of oxytocin, alone or in interplay with other hormones, in explaining variance in their parenting quality? We explored in 73 first-time fathers the development of five hormones that have been suggested to play a role in parenting: oxytocin (OT), vasopressin (AVP), testosterone (T), oestradiol (E2) and cortisol (Cort). In an extended group of fathers (*N* = 152) we examined associations with fathers’ behaviour with their 2-month-old infants. OT and E2 showed stability from the prenatal to the postnatal assessments, whereas AVP and T decreased significantly, and Cort decreased marginally. OT on its own or in interplay with other hormones was not related to paternal sensitivity. Using an exploratory approach, the interaction between T and E2 emerged as relevant for fathers’ sensitive parenting. Among fathers with high E2, high T was associated with lower sensitivity. Although we did not find evidence for the importance of OT as stand-alone hormone or in interplay with other hormones in this important phase in men's lives, the interaction between T and E2 in explaining variation in paternal behaviour is a promising hypothesis for further research.

This article is part of the theme issue ‘Interplays between oxytocin and other neuromodulators in shaping complex social behaviours’.

## 1. Introduction

Talking about adolescents, we tend to attach heavy weight to their hormones and changing hormonal levels as an explanation for their challenging social behaviors, and indeed there is some empirical basis for this widespread idea [1,2]. The same is true when pregnant women and new mothers complain about their failing memories, the so-called ‘baby brain’: hormonal changes are supposed to have affected their brains [3,4]. But what happens to men when they become fathers? Are they also subject to hormonal changes, and are their hormonal levels related to the way they interact with their infants? In the current study we explored, in first-time fathers, the development of five hormones that have been suggested to play a role in parenting: oxytocin (OT), vasopressin (AVP), testosterone (T), oestradiol (E2) and cortisol (Cort). We measured fathers' salivary hormonal levels before and after the birth of their infant. Moreover, we examined whether fathers' hormonal levels were associated with the quality of their interactive behaviour with the newborn.

Paternal hormonal levels during pregnancy are almost uncharted territory. One study measured salivary T, E2, Cort and progesterone in 29 men during pregnancy and found declines in T and E2, but no changes in men's Cort or progesterone levels [5]. Interestingly, men's larger declines in E2 and T during pregnancy were related to greater self- or partner-reported involvement in household and infant-care tasks three months postpartum [6]. Since postpartum hormones were not assessed, it is unclear whether T and E2 levels continued to decrease over the perinatal period, and whether progesterone or Cort levels showed any change from before to after the birth of the infant. Remarkably absent in most hormone studies on fathers around childbirth is OT, which is the most popular hormone related to social interaction and parenting [7]. However, in the context of childbirth, paternal OT may be considered an oxymoron as ‘oxytocin’ is derived from the Greek words for ‘swift childbirth’, and giving birth is typically not experienced as swift and certainly does not constitute the father's role in the couple's transition to parenthood.

Nevertheless, the role of OT in fathers' parenting behaviour has been demonstrated in randomized trials administering OT with a nasal spray, which enhanced paternal sensitive structuring behaviour in fathers interacting with their toddlers [8–10]. Natural variation in endogenous OT levels has also been related to paternal caregiving in a small number of studies. About half of them revealed negative or non-significant associations between plasma or saliva OT concentrations and fathers' interactive behaviors [11,12], the other half—all studies from Ruth Feldman's laboratory—reported associations indicating that fathers with higher OT concentrations showed on average more positive parenting behaviors [13]. A study measuring fathers' urinary OT three times during pregnancy (32, 33 and 34 weeks of gestation) did not find significant associations between the three time points, no differences in OT levels and no associations with paternal interest in caregiving [14].

Vasopressin is a neuropeptide that is structurally very similar to OT and has been suggested to be particularly relevant to paternal behaviour based on animal studies. In marmosets, AVP receptor density in the prefrontal cortex is higher in fathers than in non-fathers [15], and male prairie voles have elevated AVP after mating, promoting territoriality and partner protection [16]. Research in humans is relatively scarce. In one correlational study AVP was associated with fathers' stimulatory interaction with their infants [17]. In another study AVP was unrelated to fathers' neural or behavioural responses to infant threatening situations [18]. However, administration of AVP affected neural and behavioural responses of expectant fathers to sounds of crying infants [19,20], pointing to a role for AVP in responding to infant distress, one of the key requirements for sensitive caregiving. In expectant fathers, AVP administration promoted attention to virtual baby-related avatars [14]. To the best of our knowledge, no study has so far examined the development of AVP in fathers transitioning into fatherhood.

The scarcity of studies on fathers' AVP is in contrast with the relative abundance of studies on fathers' T levels. Studies using between-subject designs have pointed to lower T in fathers compared to non-fathers, as evident from two meta-analyses [21,22], and the (only) two within-subject studies following fathers from the prenatal to the postnatal phase [23,24] partly confirm this effect. Such within-subject studies are the more important as the results of Grebe *et al*. [21] highlight that part of the difference in T between fathers and non-fathers is explained by fathers being more often in a close partner relationship, which in and of itself seems related to lower T. According to the *Challenge hypothesis*, T levels are higher in the context of competition and lower in the context of monogamous relationships [25] and in the context of caregiving [26]. Results of studies relating T to parenting quality however are mixed, and the combined effect size for this association was larger in the Grebe *et al*. [21] meta-analysis (*r* = 0.33, based on 11 effect sizes, *N* = 504) than in the Meijer *et al*. [22] set of studies (*r* = 0.07, based on 18 effect sizes, *N* = 2164).

The role of E2 in paternal behaviour is far from clear. E is essential for spermatogenesis, and is mostly synthesized via peripheral aromatization of T [27], which may suggest overlapping or interaction effects with T in the associations with paternal behaviour. Studies in non-human mammals show a mixed pattern, with positive as well as negative and absent associations between E2 and parenting quality [28–30]. Among the few studies that measured E2 in fathers during pregnancy, one found a decrease in E2 [5] and the other found no change [31].

Lastly, Cort is considered relevant in the context of parenting. During labour, both fathers and mothers show substantial increases in Cort [24], perhaps best illustrating the stress accompanying the birth of an infant, also for fathers. Similarly, Cort levels have been found to increase when fathers, particularly first-time fathers, were exposed to infant crying [32], but to decrease when they held their newborn [33] or interacted with their toddler [34]. High basal Cort levels in fathers during pregnancy were related to lower quality of parenting six weeks postnatally [35]. As we have previously suggested, Cort reactivity may be functional in responding to stressors such as the birth experience or infant distress, but chronic high basal Cort levels may not be conducive to sensitive parenting [36].

Based on the literature reviewed above, we must conclude that the evidence for associations between parenting hormones and parenting behaviour in fathers is mixed at best. Some studies find associations, others not; the majority of the evidence is based on correlational research with often small samples. One explanation for inconsistent results is that bivariate associations between hormones and parenting behaviour may either be obscured or modulated by interaction effects. First, interaction effects between hormones may occur. According to the dual hormone hypothesis, T and Cort act in concert, with effects of T dependent on Cort levels [37]. In the context of fathers' parenting behaviour, only a combination of high T and high Cort levels was associated with less sensitive parenting [35,38]. T may also moderate associations between OT and parenting: in fathers with high T levels, OT was negatively associated with affectionate touch [39]. Hormones indeed act in concert on a biochemical level. T is metabolized to E2, which in turn is critical for OT synthesis [40,41]. Given the structural similarity of OT and AVP, high OT concentrations may lead to binding of OT to AVP receptors, shifting the balance between OT and AVP in the brain [42].

Second, early life experiences may influence the associations between hormones and behaviour. Early life stress was meta-analytically found to be related to lower basal OT levels, but also to affect responses to intranasally administered OT [43]. Similar effects were found for AVP administration, with effects of vasopressin on neural processing of infant cry sounds being stronger in fathers with less negative caregiving experiences [20]. Such effects may be due to experience-dependent pathways and mechanisms such as altered receptor density, affinity and methylation of genetic polymorphic pathways that regulate the endocrine system [44]. Indeed, early adversity is meta-analytically associated with higher levels of methylation of the oxytocin receptor gene *OXTR* [43].

The current study addresses two main aims regarding five hormones potentially involved in fathering, in particular OT, AVP, T, E2 and Cort. The first aim pertains to stability and change of hormonal levels in expectant fathers before and after the birth of their child. Three questions are central to this aim. First, do mean levels of the five hormones change across childbirth? Second, does the rank ordering of each of the hormonal levels in expectant fathers stay the same across childbirth? Third, do the correlations between the five hormones before childbirth remain similar compared to the pattern after the child is born? Because stability and change of hormonal levels in expectant fathers are largely unexplored no directional hypotheses are proposed, except perhaps for T where a decrease in T has been expected in the literature. The current approach should primarily be considered descriptive and exploratory.

The second aim is the search for interactions among the five hormones, or interactions between hormonal levels and adverse childhood experiences, in predicting concurrent paternal sensitive responsiveness in interaction with their two-months old infant. Here we focused in the first round on OT in relation to paternal sensitivity because of the hypothesized facilitating role of OT in fathering [7], on the possible interactions of OT with each of the other four hormones, and on the possible interactions of OT with childhood experiences, which may modulate the associations of OT with the quality of paternal interactions with the infant [43]. In the last round we explored the predictive role of interactions between any possible pairing of each of the five hormones OT, AVP, T, E2 and Cort. This inductive approach is comparable to unsupervised machine learning in that no specific hypothesis or expectation restricts the search for the most feasible prediction of the outcome variable, in this case paternal sensitive interaction with their two-month-old infant. The results of this approach should be considered grounded hypothesis for further work in independent samples.

## 2. Results

### (a) Hormonal levels: stability and change in the prenatal to postnatal period

**Figure 1.**
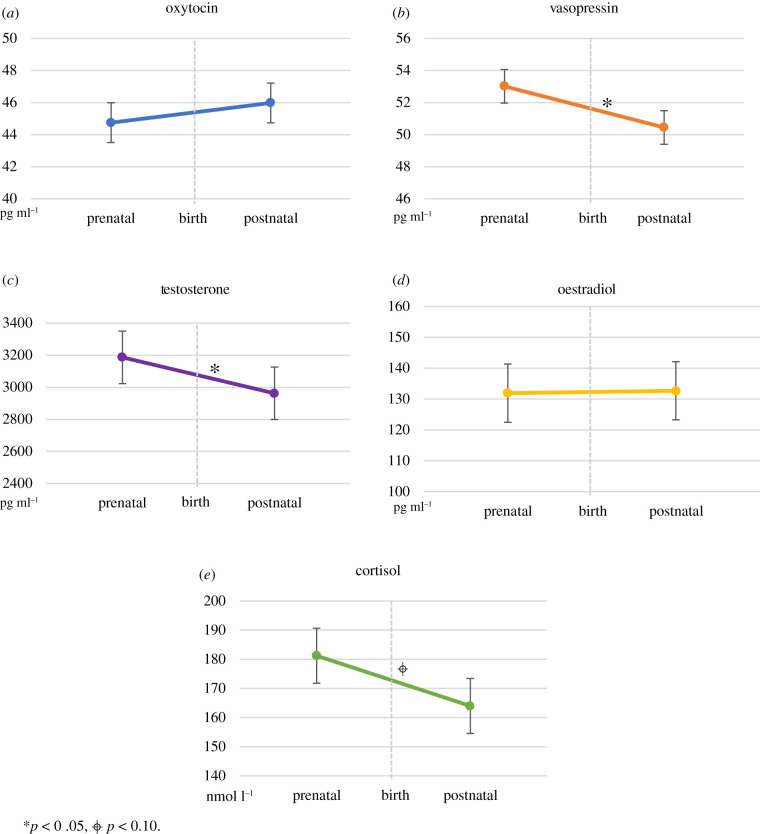
Development of oxytocin, vasopressin, testosterone, oestradiol and cortisol (M, SE) in men from before to after the birth of their first infant. *p < 0.05, ⌖ *p* < 0.10.

Figure 1 shows the development of hormonal levels from the third trimester of the pregnancy (on average four weeks before the birth of the infant) to on average five weeks after the birth of the infant. OT levels remained stable (*t*_72_ = 0.97, *p* = 0.34). Prenatal levels were moderately related to postnatal levels (*r* = 0.29, *p* = 0.01; table 1). By contrast, AVP levels decreased significantly over time (*t*_72_ = −2.15, *p* = 0.04), and no significant association was found between prenatal and postnatal AVP levels (*r* = 0.07, *p* = 0.59). Regarding the steroid hormones, T showed a significant decrease (*t*_72_ = −2.30, *p* = 0.025). Prenatal T levels were highly correlated with postnatal levels (*r* = 0.75, *p* < 0.001). Prenatal and postnatal E2 levels were also strongly correlated (*r* = 0.81, *p* < 0.001), but E2 levels remained stable in the perinatal period covered by our measurements (*t*_72_ = 0.14, *p* = 0.89). Finally, Cort levels tended to decrease over time (*t*_72_ = −1.76, *p* = 0.08). The correlation between prenatal and postnatal levels was *r* = 0.27 (*p* = 0.02; figure 1 and table 1).

**Table 1.** Associations between hormone levels before and after childbirth (*N* = 73). OT = oxytocin, AVP = vasopressin, T = testosterone, E2 = oestradiol, Cort = cortisol. Correlations in bold are significant. Correlations at the diagonal represent associations over time.

postnatal	prenatal				
	OT	AVP	T	E2	Cort
OT	**0.29**	0.06	−0.14	−0.18	−0.07
AVP	**0.66**	0.07	**0.27**	−0.03	0.13
T	−0.08	0.03	**0.75**	**0.62**	**0.26**
E2	0.13	0.10	**0.62**	**0.89**	0.11
Cort	−0.21	−0.10	0.06	−0.05	**0.26**

### (b) Hormonal levels: patterns of associations

In the prenatal phase, OT levels were not significantly associated with other hormones (table 1). Fathers' basal T levels were significantly associated with their E2, AVP and Cort levels; fathers with higher T also had higher E2, AVP and Cort. The association with E2 was particularly strong (*r* = 0.62), and remained of the same strength after the birth of their first infant. When their infants reached five weeks of age, fathers' OT levels were significantly associated with their AVP levels (*r* = 0.66), which was not the case in the prenatal phase (*r* = 0.06). Other associations were not significant and mostly remained of similar strength (table 1), although the non-significant association between OT and E2 changed from negative in the prenatal period (*r* = −0.18) to positive in the postnatal period (*r* = 0.13).

### (c) Associations with sensitive parenting at two months

In the total group of 152 fathers, no background variables were related to both hormonal levels and parental sensitivity. The only significant association with sensitivity was found for father's educational level, *r*_150_ = 0.27, *p* < 001. Fathers with higher educational levels were rated as more sensitive in interaction with their child.

#### (i) Main effects

We examined associations between OT levels and sensitive parenting at the infant age of two months. OT was not related to more sensitive parenting (*r*_150_ = −0.05, *p* = 0.56) and the partial correlation controlling for paternal education was *r*_149_ = −0.01, *p* = 0.91. None of the other hormones showed significant bivariate correlations with sensitive parenting, either (AVP: *r*_150_ = 0.04, *p* = 0.62; T: *r*_150_ = −0.08, *p* = 0.32; E2: *r*_150_ = −0.03, *p* = 0.74; Cort: *r*_150_ = 0.14, *p* = 0.08).

#### (ii) Interactions

Using the Feasible Solution Shiny Application (rFSA) for the identification of interaction terms explaining most variance of fathers' sensitivity, we found no significant main or interaction effect of OT. When OT was not forced into the regression equation, the interaction between E2 and T provided a feasible solution for the fit of the data, and the model explained a significant proportion of the variation in paternal sensitivity (multiple *R*^2^ = 0.06, adjusted *R*^2^ = 0.04, *F*_3,148_ = 3.10, *p* = 0.028).

**Figure 2.**
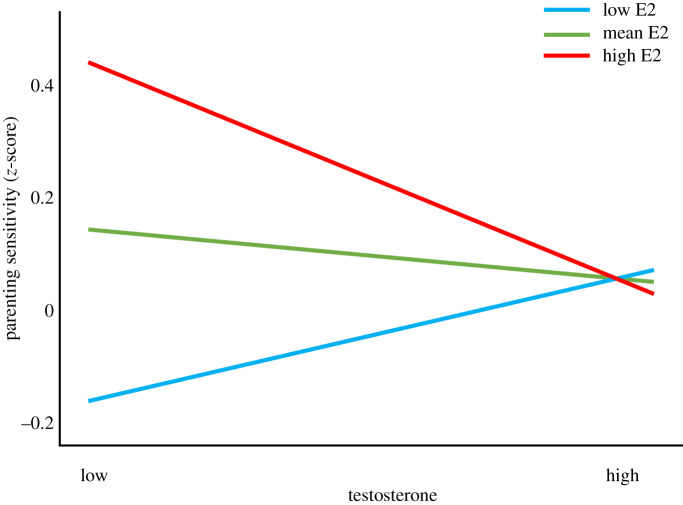
Interaction of paternal testosterone (T) and oestradiol (E2) levels in the prediction of sensitive parenting at the infant age of 2 months. Note that the green line represents the simple slope for the association between T and sensitive parenting at mean E levels, the blue line represents the simple slope for the association between T and sensitive parenting at low E2 levels (mean − 1 s.d.) and the red line represents the simple slope for the association between T and sensitive parenting at high E2 levels (mean + 1 s.d.).

A *post-hoc* multiple regression analysis including condition (experimental versus control for Expectant Fathers), hormone-related analysis batch or time between saliva sampling and observation of fathers' sensitive parenting, father's age, BMI and educational level in the first two steps confirmed the significant interaction effect of T and E2. The total model was significant (*R*^2^ = 0.13, *F*_8,143_ = 2.57, *p* = 0.012). Condition, batch and time between saliva sampling and observation of father's parenting quality had no effect. Significant predictors were education, *β* = 0.24 (*p* = 0.005) and the interaction between T and E2, *β* = −0.20 (*p* = 0.038). Analyses without the covariates showed similar results: the interaction between T and E2 predicted fathers' sensitive parenting significantly, *β* = −0.25 (*p* = 0.013). To further examine the interaction, we inspected the standardized simple slopes for the association between T and sensitive parenting at low (mean − 1 s.d.), mean, and high (mean + 1 s.d.) levels of E2. At low levels of E2, the simple slope for the association between T and fathers' sensitive parenting was *β* = 0.13, at mean levels of E2 it was *β* = −0.05, and at high levels of E2 the simple slope amounted to *β* = −0.23, implying that particularly in fathers with high E2 levels, T was negatively related to sensitivity (figure 2). It should be noted that figure 2 shows regression lines that seem to allow for two interpretations, namely that under the condition of higher E2 either higher T is associated with lower parental sensitivity, or/and lower T is associated with higher parental sensitivity. The scatterplot of the association in the sub-set of cases with the 33% of highest E2 scores, however, showed that relatively few cases were in the lower T (lower than the mean AUC of 2000 pg ml^−1^) and higher sensitivity regions (see electronic supplementary material). This makes the interpretation that among fathers with high E2, high T was associated with lower parental sensitivity more plausible.

### (d) Sensitivity analyses

The analyses were repeated excluding the six participants that did not meet the original inclusion criteria (ADHD, *n* = 3; medication use, *n* = 3) but were included to increase the sample size. The analyses excluding these six participants showed very similar results to those reported in the main analyses. OT levels remained stable over the perinatal period, and prenatal levels were moderately related to postnatal levels. OT was not related to sensitive parenting. Education and the interaction between T and E2 explained the variance in fathers' sensitive parenting. The results are presented in more detail in the electronic supplementary material.

## 3. Discussion

Of five hormones (the peptides OT and AVP, and the steroids T, E2 and Cort) potentially involved in becoming and being a father, oxytocin (OT) seemed least related to childbirth or quality of paternal interactions with the newborn in the current study. Despite its reputation in the popular literature as well as in some scientific models [44], OT levels were not found to change around childbirth nor to explain variation in paternal interactive behaviour with the newborn. We had expected to find a positive association between OT levels and sensitive parenting behaviour in fathers, although we noted that the evidence so far has come from a single laboratory, with non-replications in other studies (see for a review [13]). This would make replication of the positive results in an independent set of studies even more important. Prenatal OT predicted postnatal levels of AVP, but AVP significantly decreased after childbirth whereas in non-human species higher AVP has been shown to trigger more parenting behaviour. However, in human fathers results for the association between AVP and paternal behaviour are equivocal (e.g. [45]). By contrast, the combined role of testosterone (T) and oestradiol (E2) was most prominent. T decreased substantially from the prenatal to the postnatal period, whereas E2 levels remained the same. Although T and E2 each separately were not associated with paternal sensitivity, the interplay between T and E2 appeared to be important: in fathers with high E2, high T was associated with lower-quality paternal interactions with the newborn. Early childhood adversities did not moderate these associations.

As Hammes & Levin [46] argued, the concept of androgens as specifically ‘male’ hormones, and oestrogens as exclusively ‘female’ attributes is outdated and not in accordance with recent empirical evidence from animal and human research. Chemically, the enzyme aromatase turns androgens into oestrogens such that T is transformed into E2 via aromatase, and this is suggested to happen in various regions of the male (and female) brain as it promotes complex parenting behaviour [47]. It is also a misconception that E2 and T would only be important in the reproductive functioning of humans. Increasing evidence suggests that their impact on cognition, behaviour and emotions is considerable, and that in males T might influence these non-reproductive processes through its transformation to E2, making E2 as much a ‘male’ hormone as T [48]. The strong correlation between T and E2 levels in prenatal and postnatal assessments in the current study points at this intimate connection between the two steroids. From this perspective, the significance of the interplay between T and E2 in explaining paternal interactive sensitivity to the newborn is not surprising. In animal studies (e.g. in California mice [47,49]) it is hypothesized that paternal behaviour is related to ‘femininity’ by the aromatization of T, thus suggesting that the combination of lower T and higher E2 would predict higher levels of paternal sensitivity. In our study this combination of low T and high E2 was underrepresented, which might mean that about two months after childbirth some first-time fathers may still be in a transition to higher levels of E2 (through aromatase of T) to support full-blown paternal sensitivity.

It has been argued that human males are prepared for fatherhood by hormonal changes triggered through childbirth, not as an exclusively female biological event consisting of parturition and lactation but as a social process stimulating care by visual and olfactory cues of the newborn. In some male mammals the switch ‘from killing to caring’ seems to be turned on by pup odours inactivating the vomeronasal organ, preventing infanticide and paving the way for care based on conserved connections between regions of the parental brain [50,51]. Some decades ago, Wynne-Edwards [52] already suggested that the transition of mammalian males to fatherhood might be facilitated through similar neuroendocrinal processes to those found to be important in females, making males less aggressive and more protective to their offspring [53]. However, in species with biparental care a causal role for such processes has been difficult to find [50,54] and indirect effects may be more important.

Nevertheless, meta-analytic evidence suggests that lower T levels may accompany the switch from expectant to newborn father and to be conducive to more and better paternal care for offspring [22]. Males with a child, with more active involvement in child care or with higher parenting quality appeared to have lower T levels [21], although the associations were modest [22]. Also, the transition to fatherhood status appeared to be associated with lowering of T levels, but most studies used cross-sectional between-subjects designs and were statistically underpowered. Studies measuring T reactivity to child stimuli or cues did not show the expected effect of lowering T levels in men, despite some promising early exceptions [22].

The current study adds to the evidence base by not only documenting significantly lower T levels within the same males across childbirth but also by emphasizing the importance of examining the role of T in the context of its interplay with other hormones [35]. We found that fathers with higher T had also higher AVP, Cort and E2. The association with the latter was particularly strong, both prenatally and after the birth of their first infant, and the interaction between T and E2 seemed to be the only hormonal predictor of a substantial part of the variation in the quality of parent–infant interactions. Because T is metabolized into E2, which is critical for the synthesis of OT, it was against our expectation that fathers' OT levels were only associated with AVP postnatally. Moreover, OT did not predict paternal sensitivity to the infant, in isolation or combined with the other hormones. Thus, we were unable to shed light on the role of OT in fathering in the perinatal phase, but we were able to provide a glimpse of the potential role of T in the complex neuroendocrine feedback and feedforward system subserving similarly complex paternal behaviour in interaction with a newborn.

Of course it is difficult if not logically impossible to derive from null results the absence of the expected associations, in particular with a relatively modest sample size for detecting possibly subtle main and interaction effects of hormones on paternal sensitivity. Although our sample size of *N* = 152 is much larger than the median sample size of *N* = 58 in the set of studies of the Meijer *et al*. [22] meta-analysis on T and fathering, it still falls short of the required 500 or more participants estimated in that meta-analysis to provide sufficient statistical power for finding the expected small hormonal main effects. Interactions are even more elusive than main effects when sample size is modest. But lack of statistical power due to a limited number of observations is only one of the issues that leave room for alternative interpretations of null findings.

These issues might be summarized in the acronym ‘utosti’ [55–57]: units of observations (u), treatments (t), outcomes (o), settings (s) and time-points (ti) that all would require random selection from a universe of possible utosti components. In the current study, the ‘treatment’ of becoming a first-time father is underspecified, also because a comparison group of non-expectant fathers is lacking; the ‘outcome’ in terms of sensitive interactions is only one dimension of the complex phenotype of parental care; and hormonal levels in saliva provide only one window into the neuroendocrine system in body and brain; while methods for, e.g., measuring endogenous OT concentrations have generated considerable controversy and debate, we used a specific approach (radioimmunoassay); ‘settings' were limited to 10 min of free play with and without play materials whereas daily family life consists of many more settings calling for active paternal involvement; and ‘time-points’ were restricted to the pre- and postnatal assessment of hormonal levels and observation of paternal interactions in the early postnatal phase. The utosti choices in our study are exemplary for studies in this area, but without broader selection of utosti components alternative interpretations of null findings accumulate into a range of questions for further empirical and meta-analytic research. The search for neuroendocrine correlates of men becoming fathers and interacting with their newborn has just started.

To conclude, we did not find support for the importance of OT as stand-alone hormone or in interplay with other hormones in the process of men transitioning into fatherhood or in predicting the quality of their interactions with the newborn. We may have missed changes in OT concentrations during earlier phases of the pregnancy or later than two months after birth, or OT might indeed be an oxymoron. By contrast, T decreased substantially from the prenatal to the postnatal period and the combination of lower levels of both T and E2 was associated with higher quality of paternal interactions with the newborn. We propose that the null findings should be considered preliminary and in need of replication with various utosti choices, and that the interaction between T and E2 in explaining variation in paternal behaviour is a promising hypothesis based on our exploratory analyses. Taking into account the dearth of behavioural studies on fathers in general [36], the number of studies on hormonal correlates of paternal caregiving is a positive exception. They represent an exemplary interdisciplinary and multilevel approach to explore bridges between a complex neuroendocrine system and the multi-faceted phenotype of emerging fatherhood.

## 4. Method

### (a) Participants

Fathers were recruited via midwife practices, child healthcare centres, municipal records and (online) advertisements. We recruited among two groups: male adults who were expecting the birth of their first baby (Expectant Fathers) and male adults who recently had their first baby (i.e. infant's age approximately two months; ‘New Fathers’). The Expectant Fathers were followed from the prenatal phase to the postnatal phase, whereas the New Fathers were observed in the postnatal phase only. In other regards, the two groups were similar. All of them were cohabitating with the child's biological mother. Exclusion criteria were a psychiatric disorder, current heavy drinking, regular use of soft drugs (cannabis products), use of hard drugs (e.g. heroin or cocaine) within the past three months, and MRI contraindications (the fathers underwent MRI scanning not reported on here). Due to recruiting difficulties, we decided to include some fathers with ADHD (*n* = 3) and fathers who used medication that might interfere with the endocrine system (metformin, *n* = 1, rhinocort nasal spray, *n* = 1 and venlafaxine, *n* = 1). Fathers were 25 to 56 years old (*M* = 33.08, s.d. = 4.45) and had completed on average 8.53 (s.d. = 1.67) years of education following primary school. The vast majority of them were born in the Netherlands (94%).

Expectant Fathers (*n* = 73) and New Fathers (*n* = 79) did not differ in age, education, BMI or country of birth (Netherlands versus other), nor in the sex of their infant (table 2).

**Table 2.** Demographics of prenatally included fathers (Expectant Fathers) and postnatally included fathers (New Fathers). *p < 0.05.

	expectant fathers (*N* = 73)	new fathers (*n* = 79)
father's age in years	33.06 (3.24)	33.10 (5.36)
father's education^a^	8.79 (1.44)	8.28 (1.84)
father's BMI	24.57 (3.51)	24.79 (3.90)
father's country of birth		
Netherlands	70 (96%)	73 (92%)
other	3 (4%)	6 (8%)
infant's age prenatal wave^b^	24.95 (2.82)	—
infant's age at 2 months	2.40 (0.87)	2.67 (0.77)*
infant sex		
male	28 (38%)	42 (53%)
female	45 (62%)	37 (47%)

^a^years past primary education.

^b^gestational age in weeks.

One father was not the biological father of the child, but had been living with the biological mother since mid-pregnancy. Out of 152 children, 148 were born after at least 37 weeks of pregnancy, three were born in the 37th week of pregnancy and one was born after 30 weeks of gestation. At the two-month sensitivity observation, the mean age of the children was 2.54 months (s.d. = 0.83), with infants of New Fathers being slightly (0.27 month) older than infants of Expectant Fathers (*t*_150_ = 2.05, *p* = 0.04). To take prematurity into account, the observation of the dyad with the infant born after 30 weeks of gestation was done at 4.90 months. Approximately half of the sample were girls (54%).

### (b) Procedure

Expectant Fathers (*N* = 73) expected their first baby at the time of enrollment and were randomized to either receiving video feedback using ultrasound between the 21st and 33rd weeks of pregnancy (VIPP-PRE [58], aiming at promoting postnatal parental sensitivity and involvement) or phone calls with information on the development of their unborn child. At 36 weeks of gestation (*M* = 36.33, s.d. = 0.48) they collected saliva samples on the mornings and evenings of two consecutive days. At around five weeks after the birth of their infants (*M* = 4.63, s.d. = 1.53) they were again asked to collect saliva samples on the mornings and evenings of two consecutive days, and at two months (*M* = 2.40, s.d. = 0.87) after the infants' births the father–infant dyads were observed in a 10 min free play session at the fathers' homes.

New Fathers (*N* = 79) had a firstborn 2-month-old infant (*M* = 2.67, s.d. = 0.77) at the time of enrollment, and were randomized to either receiving a soft baby carrier or an infant seat [59]. The current study used fathers' pretest data that were collected before randomization to one of these conditions. Depending on fathers' preference and MRI contra-indications, father–infant free play was observed either at the research centre (*n* = 65) or at the fathers' homes (*n* = 14). We examined whether the differential location would be associated with variance in sensitive parenting, but we found no significant difference in observed sensitive parenting between observations that took place in the research center or at the fathers' homes (*t*_150_ = −0.30, *p* = 0.77). In both groups, questionnaires about background variables, health and medication use were completed in the weeks before or after the assessments.

### (c) Instruments

#### (i) Hormones

Studies vary in the specimen used for the determination of hormonal levels. Measurement of hormone concentrations in the brain would be ideal, but this is not possible in non-clinical human studies. The alternatives are blood, saliva and urine. Compared to urine, saliva and blood are more concordant for at least some hormones and are preferred for assessing OT in human studies [13]. Here we used saliva samples for the determination of hormone concentrations, because it enabled the participants to collect the samples at home after awakening and at bedtime. Moreover, for steroid hormones, salivary measures may be superior given their greater bioavailability. Thus, participants collected saliva twice a day (morning and evening) on two subsequent days in the 36th week of the pregnancy and five weeks after the birth of their infants (Expectant Fathers) or in the week following the two-month observation (New Fathers). Morning saliva samples were collected immediately after awakening, before drinking, eating or fathers' brushing their teeth. Evening samples were collected right before going to bed, but before teeth brushing. Fathers were requested not to eat or drink anything but water, chew gum, smoke or physically exercise in the 30 min prior to collecting the evening sample. Via an application on their smartphones they were guided through the saliva collection procedure and reported on the time of saliva collection and on their activities (e.g. eating, drinking) in the 30 min prior to sampling. They were asked to store the samples immediately after collection in their freezer. Samples were picked up by researchers at the participants' homes and transported on ice packs to the university, where they were stored in −20°C freezers until analysis.

#### (ii) Oxytocin and vasopressin

Saliva for the determination of OT and AVP levels was collected using a cotton swab (Salivette, Sarstedt). Participants were instructed to chew lightly on the swab while moving it around in their mouths. OT and AVP levels were quantified using radioimmunoassay at RIAgnosis (Sinzing, Germany). After centrifugation of the salivettes at 4°C for 30 min with *ca* 5000 g centrifugal force, 0.3 ml of saliva for the analysis of oxytocin and 0.3 ml saliva for the analysis of vasopressin were pipetted into separate vials. The detection limits of OT and AVP were in the 0.1 pg/sample range. Inter-assay and intra-assay variability was less than 10%. By contrast to plasma, saliva proteins do not interfere in the radioimmunoassay procedure. Comparing extracted with unextracted saliva samples using this method, data are almost identical (R. Landgraf, personal communication, March 5, 2020). All Expectant Fathers' saliva samples were analysed in the same batch; analyses of the New Father samples were run in two batches, for which we controlled in the analyses.

#### (ii) Testosterone, oestradiol and cortisol

Saliva for the determination of T, E2 and Cort was collected using the passive drool method. Participants collected approximately 1.5 ml saliva in a 2 ml cryogenic vial by drooling directly into the vial or using a straw-like saliva collection aid (SalivaBio, Salimetrics). T, E2 and Cort levels were quantified at Dresden LabServices GmbH (Germany) using Luminescence immunoassay (IBL International GmbH). 50, 50 and 20 µl of saliva were used for the analysis of T, E2 and Cort, respectively. The detection limits were 1.8 pg ml^−1^ for T, 0.3 pg ml^−1^ for E2 and 0.012 µg dl^−1^ for Cort. A random selection of 29% of the pre-test and post-test laboratory and home samples were assayed in duplicate and the intra-assay coefficients of variation were 4% for T, 12% for E2 and 6% for Cort. Inter-assay variability was computed from controls run at each microtiter plate and amounted to 10% or less for T, 12% or less for E2 and 8% or less for Cort.

#### (iii) Area under the curve

For all five hormones, an area under the curve (AUC) with respect to the ground [60] was calculated based on the four repeated measurements (morning 1, evening 1, morning 2, evening 2; individual missing samples were imputed based on regression equations predicting the missing values from the available samples from the same day or the same timepoint on the other day, as in [59]). The AUCs reflect the overall OT (in pg ml^−1^), AVP (in pg ml^−1^), T (in pg ml^−1^), E2 (in pg ml^−1^) and Cort (in nmol l^−1^) secretion from the morning of day 1 to the evening of day 2. Time in-between sampling moments for the calculation of the AUC was derived from the saliva collection application on the fathers' smartphones, and entered in the formula in order to account for differences in timing of saliva collection. Participants adhered to instructions not to eat or drink anything but water, brush their teeth, or physically exercise in the 30 min before sampling for more than 90% of samples, so these variables were not included as potential covariates in subsequent analyses.

#### (iv) Sensitive parenting

Fathers' interactions with their infant were observed during 10 min of free play. During the first 5 min no play material was available, after 5 min, a research assistant handed the father a bag with age-adequate toys. Fathers were instructed to play with their child as they would normally do. The father–child interaction was videotaped and sensitivity was coded using the Ainsworth scales for Sensitivity and Cooperation [61], with scores ranging from 1 (highly insensitive/highly interfering) to 9 (highly sensitive/highly cooperative). Sensitive fathers pick up the child's signals of distress or interest in a specific toy, and they respond to these signals in a prompt and adequate way, e.g. by offering the baby a desired toy that is out of reach. Insensitive fathers miss signals of disinterest or distress, e.g. they continue tickling when the baby looks away or starts to fuss. Cooperative fathers do not interfere with their children's ongoing activities, e.g. when a baby explores a rattle they may comment but do not introduce another toy. Non-cooperative fathers may move the child without an apparent reason, or force them to hold a toy and use it in a specific way. Five coders were trained to reliability with an expert coder: ICCs (single measures, absolute agreement) based on 20 videos ranged from 0.68 to 0.76 for sensitivity and 0.64 to 0.79 for cooperation. Coders were blind to participants' intervention condition. Sensitivity and Cooperation scores were significantly and highly correlated (*r* = 0.65, *p* < 0.001), and were therefore standardized to account for mean level differences and averaged into one score indicating parental sensitivity, as in [62]. Fathers in the two groups (Expectant Fathers and New Fathers) did not differ in their level of sensitivity at 2 months (*t*_150_ = 0.30, *p* = 0.76).

#### (v) Childhood caregiving experiences

Negative childhood caregiving experiences were measured with two questionnaires, the Parent–Child Conflict Tactics Scale (CTS) [63] and the Children's Report of Parental Behaviour Inventory (CRPBI) [64,65]. The CTS assessed participants' experienced childhood maltreatment by their parents. Eighteen items, concerning Psychological aggression (5 items), Physical assault (8 items) and Neglect (5 items), were rated for mother or father (the parent for whom the highest score was applicable) on a 7-point scale (0 = never, 1 = once, 2 = twice, 3 = 3–5 times, 4 = 6–10 times, 5 = 11–20 times, 6 = more than 20 times). Scores on the Psychological aggression and Physical assault scales were combined into an Abuse subscale, and mean scores on the Abuse subscale and the Neglect subscale were averaged for the total CTS score (Cronbach's *α* = 0.85).

Participants also reported on their childhood experiences of parental love withdrawal on an 11-item questionnaire, with seven items of the Withdrawal of Relations subscale of the CRPBI, and four items of the Parental Discipline Questionnaire (PDQ) [66]. The 11-item questionnaire has been previously used to measure parental love withdrawal (e.g. [42]). Participants indicated on a 5-point scale ranging from 1 (not at all) to 5 (very well) how well each statement described their parent's behaviour, for father and mother separately (e.g. ‘My mother was a person who, when I disappointed her, told me how sad I made her’). To create a parental love withdrawal score, for each item the highest score for either mother or father was taken and these highest scores were averaged across all 11 items (Cronbach's *α* = 0.90). A total score for negative childhood experiences was computed (as in [67]) by averaging the standardized CTS score and standardized love withdrawal score (Cronbach's *α* = 0.89 across all items of both questionnaires). There was no significant difference between expectant and New Fathers on negative childhood experiences, *t*_150_ = 1.87, *p* = 0.06.

### (d) Data analysis

Missing data were imputed using single imputation with the Expectation-Maximization algorithm in SPSS-28. Hormonal levels and background characteristics were used for imputation. The number of missing values was low and Little's MCAR test [68] was not significant, indicating that missingness was completely at random. The single dataset created by the E-M algorithm could be dealt with in the exploratory rFSA approach.

First, we explored the development of hormonal levels of OT, AVP, T, E2 and Cort in the group of Expectant Fathers from before to after the birth of their firstborn child using repeated measures analyses of variance controlling for condition (video-feedback or control group). No significant condition by time effects were found. Stability and change were then examined by testing for differences between prenatal and postnatal levels using paired *t*-tests, and computing Pearson's *r* for the association between prenatal and postnatal OT, AVP, T, E2 and Cort levels. Correlations among the five hormones before and after childbirth were also computed using Pearson's *r*.

Second, we tested the association between postnatal OT levels and observed sensitive parenting in the total group of Expectant and New Fathers. Since in the total group fathers' educational level was related to parenting sensitivity, we also computed the partial correlation between OT and parenting controlling for father's education [69,70]. Next, we explored whether interactions between basal hormone levels predicted sensitivity using the Feasible Solution Shiny Application available from https://shiny.as.uky.edu/mcfsa/ [71]. The application identifies interaction terms that are the most feasible solutions for explaining variance in the dependent variable, that is in fathers' sensitive parenting. The algorithm starts with a random combination of two predictors and then exchanges one of the two variables of the interaction term and the corresponding main effect. After each change, the fit of the model is evaluated ([64]; see also the R code in the package rFSA, which produced similar results). We entered the five hormones, condition and childhood caregiving experiences as predictors, prioritizing OT in the regression equation and/or interaction term. In the absence of a significant role for OT, each pairing within the set of five hormones got an equal chance to contribute to the prediction. To reduce the risk of chance findings, we limited the search to main effects and two-way interactions.

The model that was identified with the Feasible Solution Shiny Application was *post hoc* tested using multiple regression analysis with and without the covariates father's age, BMI, educational level, condition (experimental or control group in the prenatal phase) and hormone-related analysis batch or time between saliva sampling and observation of fathers' sensitive parenting included as control variables. For illustration and interpretion of the interaction we applied the SPSS macro available at http://web.pdx.edu/~newsomj/macros.htm.

## Data Availability

The data are provided in electronic supplementary material [72].
